# Mitigating Students’ Anxiety: The Role of Resilience and Mindfulness Among Chinese EFL Learners

**DOI:** 10.3389/fpsyg.2022.940443

**Published:** 2022-07-05

**Authors:** Yanfei Shen

**Affiliations:** Department of College English, Zhejiang Yuexiu University, Shaoxing, China

**Keywords:** anxiety, EFL learners, mindfulness, resilience, foreign language education

## Abstract

To manage the undesirable effect of anxiety on students, a wide scope of research has been dedicated to determining the triggers of anxiety and pedagogical interferences that can assist students with mitigating anxiety. Mindfulness is a relaxation strategy that has been related to constructive impacts when utilized as a managing technique for stress and anxiety. Originating from the construct of mindfulness, there is a multidimensional conception acknowledged as resilience as one of the notions in the Positive Psychology (PP) literature, which highlights the organizations and individuals’ strengths and self-control to comply with accidental states. Given the eminence of mindfulness and resilience in learning situations, the present study sets forth to examine the role of these constructs in alleviating English as a foreign language (EFL) learners’ anxiety. To meet this objective, 502 Chinese EFL learners took part in this research. They were asked to respond to the three questionnaires, namely resilience, mindfulness, and anxiety. To answer the research question of the study, a linear multiple regression was run and the findings demonstrated that mindfulness and resilience together could significantly predict anxiety. Consistent with the findings, some recommendations are presented concerning the implications of the present research.

## Introduction

Some studies have been done so far on the state of students’ influential factors in the procedure of language learning, where anxiety in the language class is an example of such factors (Dewaele and Dewaele, 2017; Fathi and Derakhshan, 2019). Anxiety (i.e., stress, concern, and tension) could be the most broadly researched feeling in language acquisition and alludes to the emotion of pressure and fear particularly related to foreign language settings (MacIntyre, 2017). Foreign language classroom anxiety is usually regarded as an overwhelming element that negatively impacts language acquisition (Dewey et al., 2018). In the procedure of obtaining a foreign language, students are also affected by anxiety. Whether low, moderate, or intense, foreign language learning anxiety (FLLA) is particularly known for its negative impact on the procedure of language learning which is one of the immensely investigated subjects in academic studies (Horwitz, 2017). Likewise, the manifestation of anxiety can lead to students’ inability to engage in a language, even though they are cognitively able of obtaining it (Dörnyei, 2019). Most learners with educational challenges can succeed, but their psychological activity is obstructed by a great degree of societal anxiety.

Although all university learners encounter anxiety in their individual, societal, and educational lives, the degree of anxiety and how they react to or manage it is different. Compas et al. (2001) regard coping as necessitating self-control cycles and described it as conscious volitional attempts to control feeling, intellect, conduct, physiology, and the setting as a reaction to distressing occurrences and situations. Indeed, nowadays a more tranquil life is greatly sought across the globe, mirroring the reality that numerous individuals in various communities feel the increasing wish for a more profound, purposeful, and mindful lifestyle (van Gordon and Shonin, 2018). Mindfulness is a character attribute that has been determined as an important shielding element against outer anxiety or trouble (Calvete et al., 2017; Zhou et al., 2017). Mindfulness alludes to a conscious psychological condition by emphasizing current-moment encounters (both inner and outer triggers) without judgment or response (Kabat-Zinn, 2003). Some scholars proposed that mindfulness can be characterized as a character attribute alluding to having the tendency of being mindful in ordinary life (Bao et al., 2015). That is, mindfulness naturally signifies one’s inclination to be in a mindful state over time and state (Quaglia et al., 2015).

The notion of mindfulness, possessing its origin in the Eastern pensive exercises of Buddhism, manages two simple ideas of recognition and a neutral or unbiased viewpoint in each circumstance (Hayes et al., 2004). That is, being in the condition of mindfulness is equivalent to the flexibility of the mind, which allows a person to live in the current moment and to carefully be aware of and discern new phenomena in a particular setting, without demonstrating any prejudices (Langer, 2016). Mindfulness-based interferences have attained attention in the last 10 years to assist learners with managing anxiety and enhancing their societal-emotive working (O’Driscoll et al., 2018).

In addition, due to its various kinds, mindfulness has been implemented in a vast range of fields. For instance, it has been presented cross-contextually and organized as an intermediary strategy, a mental condition, an attribute, a response-ability, or a therapeutic strategy (Van Vreeswijk et al., 2014). Interferences that are planned to improve mindfulness look to enhance learners’ attention, and psychological well-being, by utilizing meditation and other techniques to raise recognition in the current moment; to control upsetting ideas and emotions; and to accept self, others, and the world in unbiased manners (Kabat-Zinn, 2003). Moreover, coming from the condition of mindfulness, there is a multidimensional notion called resilience in the PP domain (Wang et al., 2021; Derakhshan, 2022). Also, it is mentioned that a person encounters resilience as a result of being mindful (Galante et al., 2018). Resilience does not eliminate apprehension or destroy life troubles; rather, it enables individuals to be strong at managing issues successfully, conquering troubles, and moving on with their lives (Richardson, 2002). Resilience is characterized as a person’s capability of handling extreme difficulty, bouncing back from hardship, and carrying out the obligations of life with persistence (Kim and Kim, 2017). The word resilience is employed in the setting of stress coping, highlighting the existence of viewpoints that provoke people to transform stressing life incidences (Wang et al., 2022) and it is regarded as an attribute that allows people to conquer trouble and prosper when encountering a problem and it alludes to the ability to portray resourcefulness by utilizing present inner and outer assets as a reaction to various contextual and developmental difficulties (Sharp and Jennings, 2016). The studies indicate that mindfulness enhances well-being, provokes constructive affection, and decreases symptoms associated with stress like anxiety, pain, drug abuse, and dejection (Hofmann et al., 2010; Baer and Lykins, 2011).

Among studies on college undergraduate learners, Weinstein et al. (2009) discovered that mindfulness indicated adjustive anxiety evaluations which indicated emotive health. Their outcome further proposed that the greater degree of emotive health of more mindful people could be because of their evaluative future inconveniences in non-jeopardizing ways and their fewer utilization of avoidant managing techniques. Mindfulness may inhibit learners from growing self-notions of the absence of confidence, which mainly leads to anxiety in a foreign language (Pappamihiel, 2002). Teaching mindfulness is used in different fields like neuroscience, psychology, medicine, trading, business, and nursing (Lee et al., 2012; Kearney et al., 2014). Nevertheless, this is still a relatively novel notion in the language field. Using a study to investigate learners’ use of positive reappraisal and self-constraint in highlighting language-learning conditions, Huston et al. (2011) investigated mindfulness in class situations. For instance, Charoensukmongkol (2014) discovered that the skill of dealing with one’s feelings mediates the connection between the exercise of mindfulness and overall self-effectiveness convictions. Furthermore, mindfulness has been suggested to be an attribute that lessens people’s inclination to take part in the constant deconstructive way of thinking that brings about anxiety (Gärtner, 2013; Karelaia and Reb, 2014). Since mindful people usually focus and stay in the current moment, they are less prone to contemplate things in the past or be afraid of what could take place in the future (Weick and Putnam, 2006). However, there are not sufficient inquiries supporting the role of resilience and mindfulness on learners’ anxiety in language learning contexts. Therefore, the present paper tries to fill this gap and answer the subsequent research question:

RQ: To what extent do resilience and mindfulness contribute to the Chinese EFL learners’ anxiety?

## Literature Review

### Anxiety

The notion of anxiety is multi-faceted since people who experience foreign language anxiety are prone to sense anxiety when participating in language learning and/or utilization (Horwitz, 2017). Anxiety is regarded as a normal human feeling that can be triggered by internal and external changes, unfamiliar situations, or a feeling of vagueness. That is, it is natural for most individuals to be anxious and feel under pressure, which is viewed as anxiety when coming across a particular situation that is strange (Cubukcu, 2013). Similarly, anxiety is also defined as an uncommon and extreme sense of concern and anguish, consistently described by physiological conditions, unpredictability concerning the truth and quintessence of danger, and self-unpredictability about one’s capability of managing it (Hewitt, 2011). Anxiety is delineated as fear or as pointless insomnia after determining danger, and as a condition of stress and uncertainty, certainly regarding potential worries (Yükselir and Harputlu, 2014). Generally, language anxiety is impacted by internal psychological processes, cognitive and emotive circumstances, among other things, as well as contextual requirements and the existence of others, as regarded in different time scales (MacIntyre, 2017).

Moreover, anxiety is an intrinsic mood as well as a social structure, i.e., it is co-formed by intrinsic mental procedures, cognition, and affective moods together with the needs of the conditions and the attendance of other individuals (MacIntyre, 2017). The major source of a foreign language is the sense of inconvenience that language learners feel because they have no linguistic means to authentically show and announce themselves. Announcing oneself to the universe through a problematically managed new language inherently provokes anxiety for some individuals (Horwitz, 2017). According to Aydin (2013), anxiety alludes to a troubled affective mood where a person feels hazard, experiences a lack of power, and perceives stress when encountering an anticipated hazard. In general, anxiety takes place when a person encounters inevitable conditions, which are considered as physical or psychological threats (Szyszka, 2017). Anxiety, as a regular emotive response of the subject, adjustive and significant to their persistence, is characterized as a troublesome emotion that is related to a sense of expectation of a future risk resulting in muscular tension and conditions of being alert. This is defined by a group of physiological, behavioral, and intellectual alterations that enable the subject to shield themselves against potential risks to their honesty (Pinto et al., 2015).

### Resilience

Resilience refers to an idea about the study of mind and human growth theory which is labeled as a dynamic course relating positive alteration in highly difficult conditions or the capability of people to be resilient when encountering difficulties and should be adjusted to their environment (Connor and Davidson, 2003). Due to its expressive function, individuals could take into account resilience as a vital individual differentiator when it is essential to describe through research why some people have better performance compared to others in dealing with challenges (White et al., 2010). Resilience pertains to defensive and weak mechanisms within and outside an individual that affects their adaptation to alteration and traumatic experiences that causes a dearth of homeostasis (Brewer et al., 2019; Wang et al., 2022). International students are suggested to have the resilience to be able to handle the difficulties of their new situation and adjust to it and resilient students are described based on their capability to tackle alterations. Consequently, resilience pertains to how students can get better after a difficult situation or how they cope with it (Portnoy et al., 2018). Since its origination in psychology, resilience has been characterized in various ways due to the lack of an arrangement among researchers with diverse research prominences and tactics (Fletcher and Sarkar, 2013; Xue, 2021).

Resilience is a route that focuses on the relationship between students and their environment and measures the associations among different dimensions of psychological well-being and educational success (Van der Meulen et al., 2020). It is the process, skill, and the result of an effective adaptation despite challenging or endangering situations, and the ability to resist, adapt to and recover from hardship and anxiety (Borman and Overman, 2004). Resilience is described as advancement in preference to a characteristic: an active, transmitted procedure among individuals and their environment for resources that helps to specify and adjust themselves as healthy in the face of challenges, threats, trauma, and/or routine distress (Truebridge, 2014). Resilience is regarded as a characteristic taken from a resilient construct, comprising three sets of related structures, specifically, intrinsic assets, extrinsic assistance systems, and learned approaches (Kostoulas and Lämmerer, 2018).

Besides being able to conform to challenging conditions, resilience may be comprehended as the strength to adjust to modifications in conditions (Southwick et al., 2014). That is, resilience is the potential to respond in a self-assured way to troubles or adversities in a certain condition (Masten and Reed, 2002). A resilient student grows the potential to better handle the mistakes and setbacks instead of permitting negative conditions to impede their presentation. Being resilient refers to the ability to stay alive and handle the difficult effects of stressful conditions and different difficulties faced daily (Richardson, 2002). Based on Cassidy (2015), academic resilience has three components: perseverance, reflecting and adaptive help-seeking, and negative impacts and emotional response. Perseverance means persistence, i.e., when learners have academic resilience, they must be attentive and persistent in facing their academic difficulties. Reflecting and adaptive help-seeking is the ability of learners to reflect on their abilities so that learners can seek help adaptively according to their abilities. This component involves the ability to reflect on the abilities and strengths of learners (Cassidy, 2015). The third component is the negative impact on an emotional response. This component explains how the influence of adverse occurrences can then cause emotional responses in learners. In addition, it includes explanations related to the management of anxiety and disastrous events. For example, when students have good academic resilience skills, they can avoid protracted negative emotional responses and be optimistic about the difficulties they face (Cassidy, 2015). It is shown that learners with educational resilience enjoy good relational capabilities, self-esteem regarding their capability of studying, constructive behavior toward the school, high expectations, and cultural honor (Borman and Overman, 2004).

### Mindfulness

Mindfulness has origins in long-lasting Eastern spiritual customs, in specific Buddhist philosophy that it shows how to be available in the present and how to let go of an overdependency people have when it comes to a sense of personalized identity and instead focus on an expansive link to a feeling of oneness and incorporation in all matters (Shonin et al., 2013). Mindfulness has also been theorized as not only a dispositional attribute but also a mental condition (Brown and Ryan, 2004). The former proposes that it stays constant with time. It also suggests that some people are born with a mindful attribute that is constant and lasting. The latter, however, regards it as a short-term skill that relies on an individual’s circumstances and reasons at a specific time. This viewpoint proposes that people have been or at the very least are capable of being mindful at some point (Dane, 2011). Researchers have also contended that mindfulness can be improved through interferences and exercises (Kabat-Zinn, 2003; Baer and Lykins, 2011). Mindfulness changes the educational set inside a classroom by enabling thinking, developing significance, and taking advantage of education (Li, 2022). In a wanted mindful class, the teachers help their students with rebuilding significance, thinking about the educational experience, and taking the most benefit of the educational cycle (Wang et al., 2016). According to the study carried out so far, mindfulness is an effective method for reducing worry and pressure, dealing with focus interruptions, and improving the overall psychological well-being of college students (di Pierdomenico et al., 2017; Rajabi and Ghezelsefloo, 2020). O’Donnell (2015) proposed that mindfulness has attained global interest specifically since conditions of interruption; anxiety, struggling, and absence of link are regular and harmful.

## Materials and Methods

### Participants

Five hundred and two EFL students from Zhejiang Yuexiu University in Zhejiang Province of China were invited to participate in this study. In the sample, there were 128 male (25.5%) and 374 female (74.5%) students. They are all undergraduate students, 61.95% of which are freshmen, 36.25% are sophomores and the rest are juniors and seniors. They major in E-commerce, International Economy and Trade, International Business, Financial Engineering, Taxation, Investment, Economic Statistics, Chinese Language Education, Chinese Language and Literature, and Portuguese. Among them, there were 288 Financial majors, including students majoring in E-commerce, International Economy and Trade, International Business, Financial Engineering, Taxation, Investment, and Economic Statistics.

### Instruments

#### Resilience Scale

The resilience scale built by Lereya et al. (2016) is a 40-item measure incorporating 12 subscales evaluating students’ perceptions of their attributes and shielding dimensions embedded in the environment. Each item’s frequency was estimated on a 5-point scale from 1 (never) to 5 (always). It is worth mentioning that the reliability of the questionnaire was calculated by Cronbach’s Alpha and it was 0.97.

#### Mindfulness Questionnaire

Mindfulness was evaluated with the short type of the Five-Facets Mindfulness Questionnaire (FFMQ-SF; Bohlmeijer et al., 2011). The FFMQ-SF is a 24-item confirmed survey that requests to estimate the extent to which every declaration is true for them, and items are estimated on a 5-point Likert scale (from 1 for “never or very rarely true” to 5 for “very often or always true”). It is worth noting that the reliability of the questionnaire was calculated by Cronbach’s Alpha and it was 0.89.

#### Foreign Language Classroom Anxiety Scale

The Foreign Language Classroom Anxiety Scale (FLCAS) is planned by Horwitz et al. (1986) and includes 33 items. The scale estimates the anxiety of EFL students in utilizing or studying a foreign language. Every declaration of the FLCAS is estimated utilizing a 5-point Likert-type scale ranging from 1 (strongly disagree) to 5 (strongly agree). The reliability of this scale through Cronbach’s Alpha in this study was 0.93.

### Data Collection Procedures

For questionnaire survey, Wenjuanxing, an online questionnaire platform was used in this study. This platform is popular in mainland China, and the Chinese version of the questionnaire was used for the sake of data accuracy and easy understanding for the EFL students. Prior to data collection, the students were made well informed of the research purpose and provided their willingness consent to participate in the current study according to the ethical issues of the university research ethics committee. Then we embarked on collecting the research material. The whole period of data collection lasted for 3 weeks at the university.

### Data Analysis

To study the plausible answer to the research question of the study, a linear multiple regression analysis was run after checking its related assumptions. Indeed, multiple linear regression analysis was chosen to answer the research question as there were two predicting and one predicted variables. The analysis shows the going-togetherness of each predicting variables and the predicted variable both separately and jointly. In other words, it shows to what extent anxiety can be predicted by either of resilience and mindfulness and both together. Accordingly, both the correlation coefficient (r) and the regression coefficient (beta) are estimated to answer the research question.

## Results

The study intends to estimate how resilience and mindfulness contribute to anxiety among Chinese EFL learners. Before conducting any statistical analysis to answer the research question, it is necessary to ensure instruments used to measure our variables are reliable. Table 1 shows the results of the consistency measure of Cronbach’s alpha for estimating the reliability of the three instruments of the study.

**TABLE 1:** Reliability and descriptive statistics.

	Cronbach’s alpha	Mean	Variance	Std. deviation	Minimum	Maximum	N of items	N of learners
Mindfulness	0.89	76.30	161.31	12.701	24.00	120.00	24	502
Resilience	0.97	145.62	864.43	29.401	40.00	200.00	40	502
Anxiety	0.93	97.66	370.34	19.244	20.00	100.00	33	502

As seen in Table 1, the mean score for mindfulness is 76.30 (*SD* = 12.70). The mindfulness instrument included 24 items and had Cronbach’s Alpha = 0.89, above the required value of 0.70 for reliability. The mean score for resilience is 145.62 (*SD* = 29.40). The resilience instrument included 40 items and had Cronbach’s Alpha = 0.97. The mean score for anxiety is 97.66 (*SD* = 19.24). The anxiety instrument included 33 items and had Cronbach’s Alpha = 0.93. Accordingly, all the instruments have Cronbach’s Alpha values >0.70 which ensures the reliability of the instruments.

**FIGURE 1:**
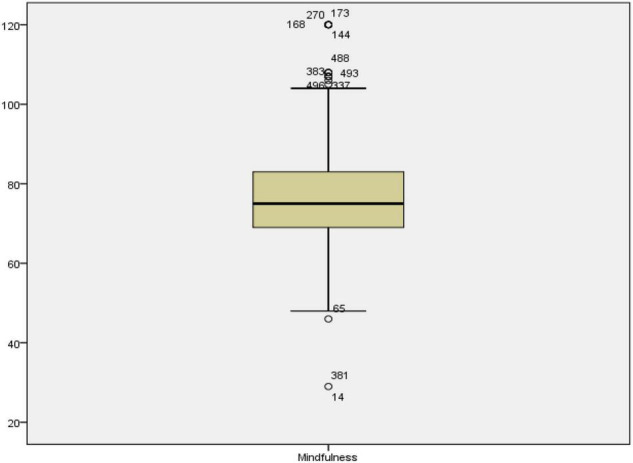
The boxplot for mindfulness.

**FIGURE 2:**
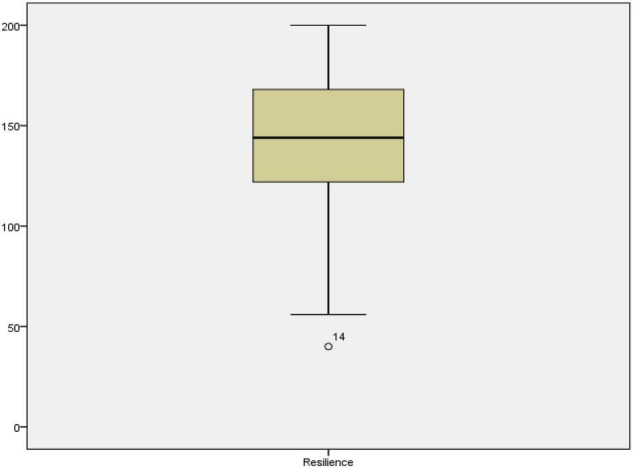
The boxplot for resilience.

**FIGURE 3:**
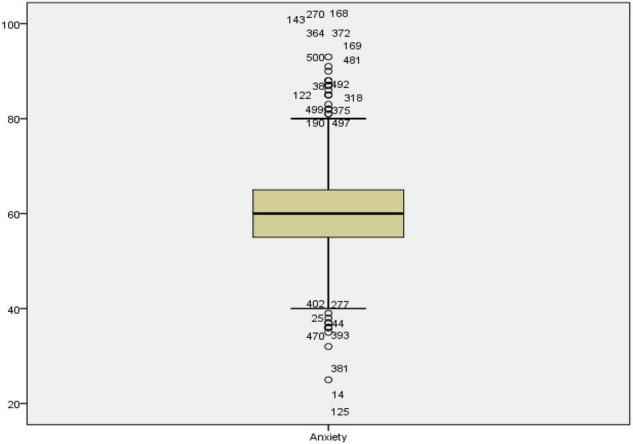
The boxplot for anxiety.

The objective of the study was to estimate how resilience and mindfulness contribute to anxiety among learners. The statistical analysis for such estimation was done through multiple regression. Anxiety was the dependent variable in the multiple regression model while mindfulness and resilience were the independent variables or predictors. Before performing regression analysis, certain assumptions needed to be met. These assumptions require that variables are continuous, the sample size is large, there is no multicollinearity, there are no outliers, data is normally distributed, relationships between predictors and dependent variables are linear, variances of residuals are the same (homoscedasticity), and residuals are independent (Pallant, 2010). The variables of the study were all interval types, and the sample size was 501, which automatically satisfied the continuity and sample size assumptions. It should be noted that a sample size >50 + 8 m (where m = number of independent variables) is suggested for multiple regression (Tabachnick and Fidell, 2007) and the sample size in the current study exceeds the recommended sample size. In the next step of the analysis, all outliers were detected and removed from the analysis pairwise. Figures 1–3 show the boxplots of the variables and the outliers.

As seen in Figures 1–3, the cases 14, 381, 65, 496, 337, 383, 493, 488, 144, 168, 270, and 173 in mindfulness, case 14 in resilience, and cases 125, 14, 381, 470, 393, 44, 25, 277, 402, 497, 190, 375, 499, 375, 318, 122, 38, 492, 481, 500, 169, 372, 364, 143, 270, and 168 in anxiety were the outliers. All these cases were removed from subsequent analysis for achieving proper statistical results. As for the multicollinearity, Tolerance and Variance inflation factor (VIF) were checked (see Table 2). The multicollinearity assumption requires that the independent variables or the predictors are not strongly correlated.

**TABLE 2:** Correlations and collinearity.

Model		Correlations			Collinearity statistics	
		
		Zero-order	Partial	Part	Tolerance	VIF
1	(Constant)					
	Mindfulness	0.389	0.390	0.390	0.949	1.053
	Resilience	0.039	−0.053	−0.049	0.949	1.053

**FIGURE 4:**
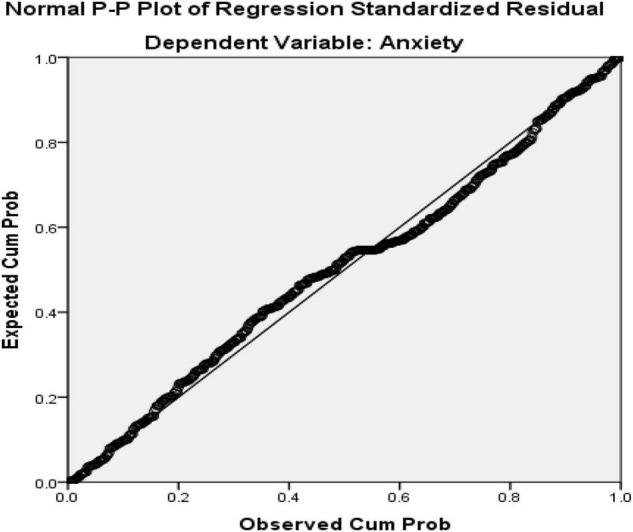
Normal Probability Plot (p-p) of the regression standardized residuals.

**FIGURE 5:**
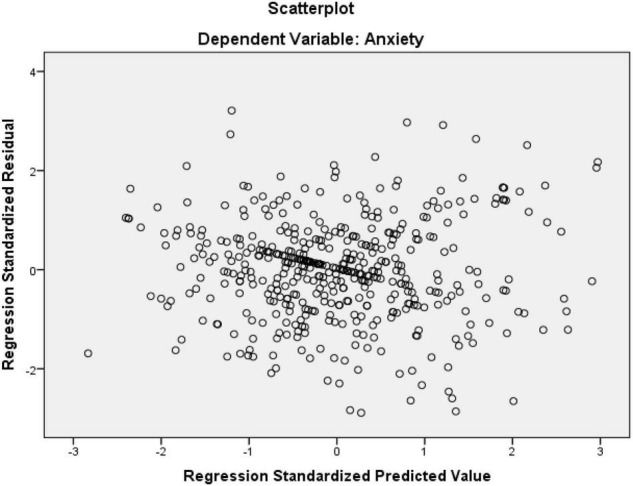
Scatter plot of residuals.

As seen in Table 2, the Tolerance values are higher than 0.10 and VIF values are less than 10 which shows no concerns for multicollinearity (Pallant, 2010). Normal Probability Plot (P-P) of the Regression Standardized Residuals and the Scatterplot were consulted to check the normality, linearity, homoscedasticity, and independence of residuals (see Figures 4, 5).

Based on the normal probability plot (Figure 4), all the points are in the shape of a straight line from the bottom left to the top right indicating the normality of the data. Additionally, residuals in the scatter plot (Figure 5) are centralized in a rectangular form with no apparent pattern that indicates linearity, homoscedasticity, and independence of residuals (Pallant, 2010).

In the next step, the regression model was consulted to check how well mindfulness and resilience together contribute to anxiety (Table 3). In the regression output, the *R*^2^ value multiplied by 100 indicates the prediction power of the model (here, mindfulness and resilience as predictors and anxiety as the dependent variable), and the ANOVA test shows the significance of the regression model.

**TABLE 3:** Coefficients.

Model		Unstandardized coefficients		Standardized coefficients	*t*	Sig.
		B	Std. error	Beta		
1	(Constant)	37.028	3.044		12.165	0.000
	Mindfulness	0.333	0.036	0.400	9.141	0.000
	Resilience	−0.016	0.014	−0.051	−1.155	0.000

Based on regression output presented in Table 4, mindfulness and resilience could predict 15% of the variance (*R*^2^ = 0.153) in anxiety which was significant (*f* = 42.20, *P* = 0.00). In other words, mindfulness and resilience together could significantly predict anxiety for 15%.

**TABLE 4:** Regression model and the associated ANOVA.

Model	*R*	*R* ^2^	Adjusted *R*^2^	Std. error of the estimate	*F*	Sig.
1	0.392^a^	0.0153	0.150	8.219	42.207	0.000^b^

*^a^Predictors: (Constant), mindfulness, resilience.*

*^b^Dependent variable: anxiety.*

Mindfulness and resilience together could significantly predict anxiety. As seen in Table 3, the Beta value for mindfulness was 0.4, *t* = 9.14, *p* < 0.01, and resilience had a Beta value of 0.05 which was less than that of mindfulness but also significant, *t* = 1.15, *p* < 0.01.

## Discussion

The current study tries to inspect how mindfulness and resilience contribute to anxiety in learners. The study included 501 participants whose mindfulness, resilience, and anxiety were measured using highly reliable questionnaires. The data analysis was done using multiple regression and displayed that mindfulness and resilience together could significantly predict anxiety for 15%. Both mindfulness and resilience could uniquely and significantly contribute to anxiety. In this section, it is discussed how theoretical explanations justify the findings and how they contribute to foreign language education. Indeed, the findings promote mindfulness and resilience as tools for dealing with anxiety in foreign language classrooms. Recent studies show that the role of emotion, including anxiety, is gaining more prominence in language learning (e.g., Boudreau et al., 2018; Teimouri et al., 2019). Anxiety is the most regularly researched feeling in foreign language education and is characterized as the concern and deconstructive emotive response triggered when studying or utilizing a foreign language (MacIntyre, 2017). Anxiety is accompanied by various symptoms in contrast with signs of mindfulness and resilience. Anxiety is associated with distraction, tension, high heart rate, sweating, etc. (Spielberger and Reheiser, 2009; Leigh, 2015; Norton and Abbott, 2016), while mindfulness has related to cognitive flexibility, focus, and impartiality (Langer, 2016).

The current study’s findings can also be discussed from an educational perspective. To put it simply, the findings promote mindfulness and resilience as tools for dealing with anxiety in foreign language classrooms. Foreign language learning experts have always suggested that teachers seek ways to diminish anxiety in language classrooms (Horwitz, 2017). Anxiety has been looked at as a big risk factor in foreign language learning and has been mentioned as a robust predictor of achievement or failure for language learners (MacIntyre, 2017). Anxiety could have various sources, including fear of negative evaluation in the classroom and personality traits (Cooper and Brownell, 2020). Raising students’ awareness about their anxiety and its impact on their performance, using group-based activities, and game activities have been suggested as techniques to reduce anxiety in the classroom. However, few studies if any have approached anxiety reduction in foreign language classrooms from mindfulness and resilience promoting perspective. Due to its various types, mindfulness could be employed in different fields. For example, meditation techniques, skills for controlling reactions, and managing the mind could be employed as ways to increase mindfulness (Van Vreeswijk et al., 2014).

The outcomes of this research agree with Palmer and Rodger (2009) who proposed that mindfulness could elevate a person’s capability to regulate anxiety, due to the outcome that one’s with a great degree of mindfulness had a much lower score on the anxiety. Anxiety pertains to fear of failure, and learners who are brave in the face of failure will outperform in difficulties (Martin and Marsh, 2020). Researchers have also contended that mindfulness can be improved through interferences and exercises. Mindfulness can keep learners from building self-conceptions about the absence of self-esteem, which is among the primary causes of language anxiety (Horwitz, 2017). These results are consistent with the former inquiries indicating the advantages of mindfulness in assisting people to do efficiently in stressful conditions (Hofmann et al., 2010). Great degrees of mindfulness are positively related to elevated emotive control skills (Brown et al., 2007), and so mindfulness can help restrain deconstructive experiences and feelings EFL students are distressed with, as well as those connected to exam anxiety, association concern, and fear of deconstructive assessment. These outcomes can also be described because mindfulness can reduce contemplation, which is connected to anxiety and sadness and is alluded to as ongoing, deconstructive, and self-oriented thoughts regarding what is to come or what has gone by Coffey et al. (2010). The results are in accordance with prior research that has revealed a relationship between mindfulness and stress reduction (Shapiro et al., 2007; Walach et al., 2007).

It is contended that improved mindfulness could enable people to encounter less anxiety by acting more congruently in their settings, and thus, be better capable of satisfying their demands and acting consistently with their principles. The results are therefore consistent with previous research carried out by Erbe and Lohrmann (2015) who indicated that mindfulness reliably had noteworthy results for its constructive effects and they concluded that mindfulness could reduce hopelessness and anxiety, in addition to stress, emotion regulation, and well-being. Also, the result supports the study conducted in Iran by Fallah (2017) who proved the effect of mindfulness on English language anxiety and he revealed that greater degrees of mindfulness were greatly associated with less English language anxiety. This was maintained by Charoensukmongkol (2014) who found an association between practicing mindfulness and language anxiety reduction. The outcome that learners who scored high in mindfulness encountered less discerned anxiety and were prone to take part in a logical, adjustive managing is consistent with the idea in self determination theory (SDT) that people with a high degree of mindfulness, or cognizance of their inner and outer encounter, are more prone to act in ways that are along with their principles. Therefore, they are more prone to adjust to inconveniences in their setting, control their feelings, and satisfy their demands (Brown and Ryan, 2004). It can be inferred that by exercising mindfulness, learners can build reflexivity and abilities that assist them with coping with anxiety, and so, helping with their involvement in highly demanding expert practices.

Furthermore, resilience is referred to as tolerability, responsibility, and perseverance (Kim and Kim, 2017). In other words, while anxiety raises concerns, apprehension, and distraction, mindfulness and resilience promote relaxation, focus, and perseverance. Therefore, theories of anxiety, resilience, and mindfulness support the finding that mindfulness and resilience can predict anxiety. Additionally, techniques to boost resilience may also deal with anxiety in the classroom. For instance, setting more manageable goals and teaching motivation-raising skills could be used to train learners to be more resilient and immune to language anxiety. Resilience is present when the individual utilizes psychological cycles and conduct in advancing individual resources and shielding oneself from the possible deconstructive impacts of inconveniences (Heshmati, 2021). Moreover, the outcomes of the present study are congruent with some other studies in other subject fields; for example, Johnston-Wilder et al. (2014) in their study indicated that learners with more degrees of resilience could manage the traumatic and demanding circumstances efficiently and they experience mathematical anxiety less. Correspondingly, research carried out by Trigueros et al. (2020) with college learners indicated that learners with great levels of resilience were less anxious when dealing with a test. Resilience is maintained to eliminate the deconstructive impacts of worry and to improve an individual’s health. Undoubtedly, scholars have discovered that resilience has an impact on unfavorable mental results, like anxiety, sadness, and post-traumatic stress disorder. Additionally, resilience could be a significant element in lessening conditions of sadness, internalizing issues, externalizing issues, and reducing overall mental worry, which assists people with sustaining healthy and constant mental conditions (Dray et al., 2017).

## Conclusion and Implications

Grounded on the upshots of the study, it is evidenced that mindfulness and resilience are mental powers that can alleviate learners’ anxiety. To decrease students’ language anxiety, both educators and learners must act in some ways. Indeed, the findings in the present research are according to what learners can do to mitigate their anxiety but include recommendations for educators. Because recognizing what to do to decrease tension in the class is a basic problem for many educators, becoming aware of such constructs, namely resilience, and mindfulness could be a reasonable necessity in this domain. Academic organizations and governments might make plans to assist learners with being more resilient, thereby reducing educational isolation and educational anxiety.

Indeed, the results of this research highlight the prominence of encouraging mindfulness and resilience in procedures of learning and education. Since an efficient academic system should guarantee the lessening of students’ anxiety during learning and offer learners higher success, being mindful and resilient need to be practiced in academic settings. The significance of resilience in educational settings is because the specific learners who encounter distressing and troubling circumstances become stronger without building any mental syndrome (Robertson et al., 2017). Accordingly, resilience has been positively related to emotive intelligence, inner inspiration, and educational presentation (Trigueros et al., 2020). Contrastingly, resilience has been negatively associated with apprehension, exam anxiety, and exhaustion (Van Hoek et al., 2019).

In addition, students may enjoy the integration of mindfulness into the educational setting, because mindfulness exercises can help teachers cope with their stress. Similarly, mindful, and resilient learners tend to have higher engagement and participation in academic activities. Indeed, support exists for designing plans for language students that promote resilience and mindfulness leading to the improvement of their ability to effectively manage the challenges that they face in the process of their learning. Additionally, Mindfulness can assist individuals to better react to mental suffering as opposed to responding in a maladjusted or overindulging way (Roemer et al., 2015; Bajaj and Pande, 2016). People with a great degree of mindfulness overall possess constructive and hopeful manners toward themselves and the future, and can better manage troubles and psychological suffering, like anxiety and sadness.

Considering the scarcity of studies on the structures of constructive affections and mindfulness, the present research increases the attention to comprehending how mindfulness is employed as an instrument for changing and maintaining constructive experiences. The outcomes of the present study have significant suggestions for those interested in planning interferences with the specific objective of improving mindfulness to reduce students’ anxiety. Particularly, research results can be applied to the theory of broaden-and-build as they recommend that mindfulness generates constructive affections like happiness as well as optimism that results in less stress.

By familiarizing students with mindfulness activities, learners can get higher awareness regarding the psychological-physical link that can also provide more mindful well-being options. Such competencies are useful not only to manage the routine stressing factors of university life but are competencies throughout life that can help them during challenging transitions and disasters throughout life in different conditions like the place of work, learning settings, family conditions, and social environments. Higher institutional support needs to be provided for mindfulness classes so that they are highly accessible, conveniently available, and even obligatory for the whole learners. Mindfulness assists the improvement of constructive affections that support the modification of negative affections like apprehension, regret, and anxiety. Such constructive affections may assist in creating individual reservoirs that result in higher life appreciation, and further meaning for life, and help shape a protecting defense to address the future problems that is in direct relation with resilience.

Syllabus designers are extremely suggested to integrate content materials that stimulate and encourage mindfulness followed by resilience. Indeed, diverse types of treatments and interventions can be designed to enhance mindfulness and resilience that bring about a reduction in learners’ anxiety. Finally, it should be noted that the current study, like many empirical studies, is not without limitations. In the present study, the researcher relied on surveys and quantification to capture the associations between anxiety, resilience, and mindfulness. Therefore, it lacks a deep description of the connection between anxiety and resilience and between anxiety and mindfulness. It is recommended that future studies include descriptive accounts of the foreign language learners’ status of anxiety, mindfulness, and resilience and explore how individuals seek unique ways to deal with their anxiety and what are the obstacles and challenges to their mindfulness and resilience. The target of the present research was merely English language college learners. Despite the dearth of studies in other areas, more investigation is desired in education to examine the connection among some issues beneficial to learning at various degrees of EFL learning, at private organizations, and even among English educators. Further research is suggested to investigate the conformity among the variables investigated in the present research concerning language success. Furthermore, in the present research, the researcher investigated the relationship among the constructs quantitatively. Both the qualitative and quantitative methods can be used to do a more precise study. Selecting several other methods, especially observation or qualitative approaches, can provide a comprehensive view of the relationship between mindfulness and resilience in EFL learners’ anxiety. Also, a longitudinal design study will be done in further studies to evaluate the long-lasting impacts of mindfulness on mitigating EFL learners’ anxiety. The participants of the study were selected only from one university, which may influence the generality of the research findings, so more research can be carried out in future among different universities and in different settings.

## Data Availability Statement

The original contributions presented in this study are included in the article/supplementary material, further inquiries can be directed to the corresponding author/s.

## Ethics Statement

The studies involving human participants were reviewed and approved by the Zhejiang Yuexiu University Ethics Committee. The patients/participants provided their written informed consent to participate in this study.

## Author Contributions

The author confirms being the sole contributor of this work and has approved it for publication.

## Conflict of Interest

The author declares that the research was conducted in the absence of any commercial or financial relationships that could be construed as a potential conflict of interest.

## Publisher’s Note

All claims expressed in this article are solely those of the authors and do not necessarily represent those of their affiliated organizations, or those of the publisher, the editors and the reviewers. Any product that may be evaluated in this article, or claim that may be made by its manufacturer, is not guaranteed or endorsed by the publisher.
